# Differential regulation by AMP and ADP of AMPK complexes containing different γ subunit isoforms

**DOI:** 10.1042/BJ20150910

**Published:** 2016-01-05

**Authors:** Fiona A. Ross, Thomas E. Jensen, D. Grahame Hardie

**Affiliations:** * Division of Cell Signalling & Immunology, College of Life Sciences, University of Dundee, Dundee DD1 5EH, Scotland, U.K.; † Department of Nutrition, Exercise and Sports, University of Copenhagen, Universitetsparken 13, 2100 Copenhagen, Denmark

**Keywords:** adenine nucleotides, adenosine 5′-diphosphate (ADP), adenosine 5′-phosphate (AMP), adenosine 5′-phosphate (AMP)-activated protein kinase (AMPK), adenosine 5′-triphosphate (ATP)

## Abstract

The γ subunits of heterotrimeric AMPK complexes contain the binding sites for the regulatory adenine nucleotides AMP, ADP and ATP. We addressed whether complexes containing different γ isoforms display different responses to adenine nucleotides by generating cells stably expressing FLAG-tagged versions of the γ1, γ2 or γ3 isoform. When assayed at a physiological ATP concentration (5 mM), γ1- and γ2-containing complexes were allosterically activated almost 10-fold by AMP, with EC_50_ values one to two orders of magnitude lower than the ATP concentration. By contrast, γ3 complexes were barely activated by AMP under these conditions, although we did observe some activation at lower ATP concentrations. Despite this, all three complexes were activated, due to increased Thr^172^ phosphorylation, when cells were incubated with mitochondrial inhibitors that increase cellular AMP. With γ1 complexes, activation and Thr^172^ phosphorylation induced by the upstream kinase LKB1 [liver kinase B1; but not calmodulin-dependent kinase kinase (CaMKKβ)] in cell-free assays was markedly promoted by AMP and, to a smaller extent and less potently, by ADP. However, effects of AMP or ADP on activation and phosphorylation of the γ2 and γ3 complexes were small or insignificant. Binding of AMP or ADP protected all three γ subunit complexes against inactivation by Thr^172^ dephosphorylation; with γ2 complexes, ADP had similar potency to AMP, but with γ1 and γ3 complexes, ADP was less potent than AMP. Thus, AMPK complexes containing different γ subunit isoforms respond differently to changes in AMP, ADP or ATP. These differences may tune the responses of the isoforms to fit their differing physiological roles.

## Abstract (precis)

AMPK complexes containing γ1, γ2 or γ3 subunit isoforms were generated by expression in human cells. They displayed differences in all three effects by which adenine nucleotides cause regulation, i.e. in allosteric activation, promotion of phosphorylation and inhibition of dephosphorylation

## INTRODUCTION

AMP-activated protein kinase (AMPK) is expressed ubiquitously and is activated by increases in AMP and/or ADP relative to ATP, thus acting as a sensor of cellular energy status [1–3]. Once activated by cellular stress it acts to restore energy homoeostasis by switching on ATP-producing catabolic pathways, while switching off anabolic pathways and other ATP-consuming processes. AMPK occurs universally in eukaryotes as heterotrimeric complexes comprising a catalytic α subunit and regulatory β and γ subunits; in humans, all three subunits are encoded by multiple genes, i.e. *PRKAA1* and *PRKAA2*, encoding α1 and α2; *PRKAB1* and *PRKAB2*, encoding β1 and β2; and *PRKAG1*–*PRKAG3*, encoding γ1, γ2 and γ3 [4–6]. All 12 possible combinations of subunits can form complexes when co-expressed [7], although certain combinations appear to be favoured in specific tissues [8]. AMPK complexes are activated by upstream kinases that phosphorylate a conserved threonine residue (Thr^172^ in rat α2 [9]) within the activation loop of the α subunit kinase domain. Upstream kinases that phosphorylate Thr^172^ include the tumour suppressor LKB1 (liver kinase B1) [10–12] and the Ca^2+^/calmodulin-dependent kinase, calmodulin-dependent kinase kinase (CaMKK)β [13–15]. The β subunits contain conserved domains called carbohydrate-binding modules (CBMs), related to domains found in enzymes that bind starch or glycogen. One surface of the CBM causes cellular AMPK complexes to bind to glycogen particles [7,16,17], whereas the opposite surface associates with the N-terminal lobe of the kinase domain, with the cleft between them forming the binding site for synthetic activators of AMPK, such as 991 and A-769662 [18] and the natural activator salicylate [19,20]. A-769662 can cause a dramatic allosteric activation (>60-fold) of AMPK even when it is not phosphorylated on Thr^172^ [21]. The γ subunits of AMPK contain four tandem repeats of a sequence motif termed a cystathionine-β-synthase (CBS) repeat [22,23]. These assemble in a pseudo-symmetrical manner to form a disc-like shape with one repeat in each quadrant, generating two potential ligand-binding clefts between repeats 1 and 2 and two between repeats 3 and 4 [24] (binding sites are numbered according to the CBS repeat interacting with the ribose ring of the bound nucleotide [25]). However, one site (site 2) appears to be unused, whereas another (site 4) is thought to be permanently occupied by AMP [24] (although this has been challenged [26]). The remaining two (sites 1 and 3) bind AMP, ADP or ATP in a competitive manner [24,27].

AMP binding activates AMPK by three effects: (i) promotion of Thr^172^ phosphorylation by LKB1; (ii) inhibition of Thr^172^ dephosphorylation by protein phosphatases; and (iii) allosteric activation [28]. Binding of ATP inhibits AMPK by antagonizing the binding of AMP without causing any of the activating effects, whereas binding of ADP has been reported to mimic two of the effects of AMP, i.e. (i) [29] and (ii) [27], although not allosteric activation (iii). Using native AMPK purified from rat liver, our group recently reported that ADP mimicked the effect of AMP to inhibit Thr^172^ dephosphorylation [effect (ii)], but we were unable to discern any effect of ADP on Thr^172^ phosphorylation [effect (i)]. We also found that AMP was almost 10-fold more potent than ADP in inhibiting Thr^172^ dephosphorylation, suggesting that AMP may remain as the crucial signal [28].

Although the existence of multiple isoforms of the AMPK-α, -β and -γ subunits has been known for many years [4–6], the question of whether the different isoform combinations have distinct functions remains only partially answered. The α2 subunit is found in various cell types to be more enriched in the nucleus than the α1 subunit [30–32], although both contain well-defined nuclear export signals at their C-termini [33]. The CBM of the β2 subunit appears to have a higher affinity for glycogen than that of β1 [34] and to bind more tightly to glycogen particles in intact cells [7]. The greatest sequence variability occurs within the γ subunit isoforms; γ2 and γ3 contain long N-terminal extensions that are unrelated to each other and are absent from γ1, although both can also occur as shorter variants due to the use of different transcriptional start sites [35,36]. Although the exact functions of these N-terminal extensions remain unclear, it is tempting to propose that they target AMPK complexes to different subcellular locations, and there is indeed some evidence that different isoform combinations are found at different locations [30,35,37]. The γ3 isoform is only thought to be expressed significantly in skeletal muscle [5,36], where it forms a complex preferentially with the α2 and β2 isoforms [8]. Previously, we reported that the AMPK activator PT-1 activates γ1- but not γ3-containing complexes in mouse skeletal muscle, whereas the nucleoside AICAR (5-aminoimidazole-4-carboxamide riboside), which is taken up into cells and converted into an AMP analogue [38], activated both isoforms. In contrast with AICAR, PT-1 did not promote glucose uptake, consistent with previous findings in knockout mice suggesting that the γ3 complex is required for effects of AMPK on glucose uptake [39]. These results initially suggested that PT-1 was a selective activator of AMPK complexes containing the γ1 rather than the γ3 isoform. However, when we stably expressed FLAG-tagged human γ1, γ2 or γ3 in HEK-293 cells and immunoprecipitated the different γ isoform complexes using anti-FLAG antibodies, we found that all three complexes were activated by PT-1. We also found, in contrast with previous proposals [40], that PT-1 activated AMPK by inhibiting the respiratory chain and thus increasing cellular AMP. Thus, the selectivity for γ1 over γ3 complexes in skeletal muscle must be a specific feature of that cell type, most probably because PT-1 does not increase AMP in the cell compartment in which γ3 complexes are located.

Having constructed human embryonic kidney (HEK)-293 cells expressing FLAG-tagged human γ1, γ2 or γ3 for this previous study, we decided to make use of them to examine whether there was any differential regulation of AMPK complexes containing different γ subunit isoforms by adenine nucleotides.

## EXPERIMENTAL

### Materials and proteins

Berberine chloride, phenformin and A23187 were from Sigma–Aldrich. A769662 was synthesized as described previously [41]. Recombinant human CD73 was from R&D Systems. Human CaMKKβ was expressed as a GST-fusion and purified from *Escherichia coli* [15]. Human LKB1–STRAD (Ste20 related adaptor)–MO25 (mouse protein-25) complex was expressed in an insect cell baculovirus system [42]. PP2Cα was expressed in *E. coli* and purified as described in [43]. Protein phosphatase-1 catalytic subunit (PP1γ) was expressed in *E. coli* as a GST-fusion protein. Polycistronic bacterial expression plasmids encoding the human α1β1γ1, α2β2γ1 and α2β2γ3 complexes of AMPK were gifts from the Division of Signal Transduction Therapy (University of Dundee), AstraZeneca and Pfizer respectively. They were purified using the N-terminal His_6_-tags on the α subunits, as described previously [19].

### Antibodies

The anti-pThr^172^, -β1, -pan-α and -glyceraldehyde-3-phosphate dehydrogenase (GAPDH)  antibodies were from Cell Signaling Technology. Antibodies against the FLAG epitope were from Sigma–Aldrich. The anti-γ1 antibody was from Abcam and the anti-α1, -α2 [44], -β2 [5], -γ2 and -γ3 [6] antibodies were described previously. Secondary anti-rabbit and anti-sheep immunoglobulin antibodies were from Li-Cor Biosciences.

### Generation of stable cell lines expressing human γ1, γ2 and γ3

HEK-293 cells expressing tetracycline-inducible human γ1, γ2 and γ3 with N-terminal FLAG tags were obtained by inserting the appropriate DNA into cells carrying a Flp recombinase target site with co-transfection of DNA encoding Flp recombinase; expression of the γ subunits was induced by adding tetracycline (1 μg·ml^−1^) to the medium for 48 h [39,45].

### Cell culture

HEK-293 cells were grown in Dulbecco's modified Eagle's medium (DMEM) containing 4.5 g/l glucose, 10% (v/v) FBS, 100 units/ml penicillin, 100 μg/ml streptomycin, 200 μg/ml hygromycin and 15 μg/ml blasticidin. The medium was replaced with medium containing 1 g/l glucose, 16 h before the treatments described in the text.

### AMPK assays in cell lysates

Cell lysates were made using the rapid lysis procedure [46]. Lysates containing stably expressed recombinant FLAG-tagged γ subunits were immunoprecipitated from HEK-293 cell lysates (90 μg of lysate protein) by incubation at 4°C for 2 h on a roller mixer with 7.5 μl of EZview Red anti-FLAG M2 affinity gel (Sigma–Aldrich). After extensive washing, the immunoprecipitates (IPs) were assayed for AMPK activity on an orbital shaker as described previously [46,47] except that the *AMARA* peptide substrate [48] was used in all assays apart from those shown in Figures 3 and 4. The SAMS peptide was used in the latter because, for reasons that remain unclear, the degree of allosteric activation is always greater using this substrate.

### Allosteric activation of immunoprecipitated γ complexes

AMPK was activated by incubating the cells in phenformin (10 mM) for 60 min prior to cell lysis. Cell lysates (360, 360 and 560 μg of lysate protein for γ1, γ2 and γ3 respectively) were incubated with 50 μl of EZview Red anti-FLAG M2 affinity gel and immunoprecipitated and washed as described previously [49]. The final precipitates were resuspended in 2 ml of Hepes assay buffer (50 mM sodium Hepes, pH 7.4, 1 mM DTT, 1 mM EDTA and 0.02% Brij-35) and divided into 20 100-μl aliquots. AMPK activity was assayed for 10 min using SAMS peptide, with concentrations of AMP and ATP specified in figures and figure legends. MgCl_2_ was kept at a constant 4.8 mM excess over the concentration of ATP.

### Promotion of activation and Thr^172^ phosphorylation by AMP and ADP

Cell lysates (140, 500 and 2000 μg of lysate protein for γ1, γ2 and γ3 respectively) were incubated with 100 μl of EZview Red anti-FLAG M2 affinity gel, immunoprecipitated and washed as described previously [49]. The IPs were resuspended in 100 μl of Hepes assay buffer and incubated in an orbital incubator with PP1γ (55 μg in 10 μl) in Hepes assay buffer for 30 min at 30°C. The dephosphorylation reactions were stopped by washing the IPs three times in Hepes assay buffer. The precipitates were then resuspended in 1 ml of Hepes assay buffer [46] and divided into 100-μl aliquots. After centrifugation, 75 μl of each supernatant was removed, 5 μl of okadaic acid (final concentration 25 μM) was added and incubation continued for 5 min at 30°C. After this, AMP or ADP and LKB1 were added as specified and the reaction started by adding a mixture of MgCl_2_ (final concentration 9.8 mM) and ATP (final concentration 5 mM). Incubation was for 10 min at 30°C, after which the reactions were stopped by washing the IPs three times in Hepes assay buffer. They were resuspended in 400 μl of assay buffer and split for assays and analysis by Western blotting.

### Protection against inactivation and Thr^172^ dephosphorylation by AMP and ADP

AMPK was activated by incubating the cells in phenformin (10 mM) for 60 min prior to cell lysis. Cell lysates (225, 225 and 455 μg of lysate protein for γ1, γ2 and γ3 respectively) were incubated with 100 μl of EZview Red anti-FLAG M2 affinity gel beads at 4°C for 2 h on a roller mixer. The beads were washed twice with 1 ml of immunoprecipitation buffer and twice with 1 ml of Hepes assay buffer [46], resuspended in 1 ml of assay buffer and divided into 100-μl aliquots. The beads were recovered by centrifugation and 75 μl of the supernatant removed. In a final volume of 50 μl, AMP, ADP (as specified), ATP (5 mM) and PP2Cα (1.5 μg) were added and the reaction was started by adding MgCl_2_ (9.8 mM) to activate PP2Cα; the reactions were then conducted in a shaking incubator at 30°C for 10 min. They were stopped by washing the beads three times in assay buffer, resuspending them in 400 μl of assay buffer and dividing them into aliquots for kinase assays and analysis by Western blotting.

### Western blotting and other analytical procedures

For analysis of proteins, SDS/PAGE was performed using precast Novex NuPAGE Bis-Tris 4–12% gradient polyacrylamide gels in the MOPS buffer system (Invitrogen). Proteins were transferred on to nitrocellulose membranes using the IBlot system (Life Technologies) and blocked in Li-Cor Odyssey blocking buffer for 1 h, and detection was performed using the appropriate secondary antibody coupled to IR680 or IR800 dye. Membranes were scanned using the Li-Cor Odyssey IR imager. For analysis of immunoprecipitations, membranes were sensitized using SuperSignal Western Blot Enhancer (Thermo Scientific) before blocking with Li-Cor Odyssey blocking buffer.

### Statistical analysis

Statistical significance was tested using GraphPad Prism 6, using tests for significance as specified in figure legends.

## RESULTS

### Characterization of cell lines expressing human γ1, γ2 or γ3

**Figure 1:**
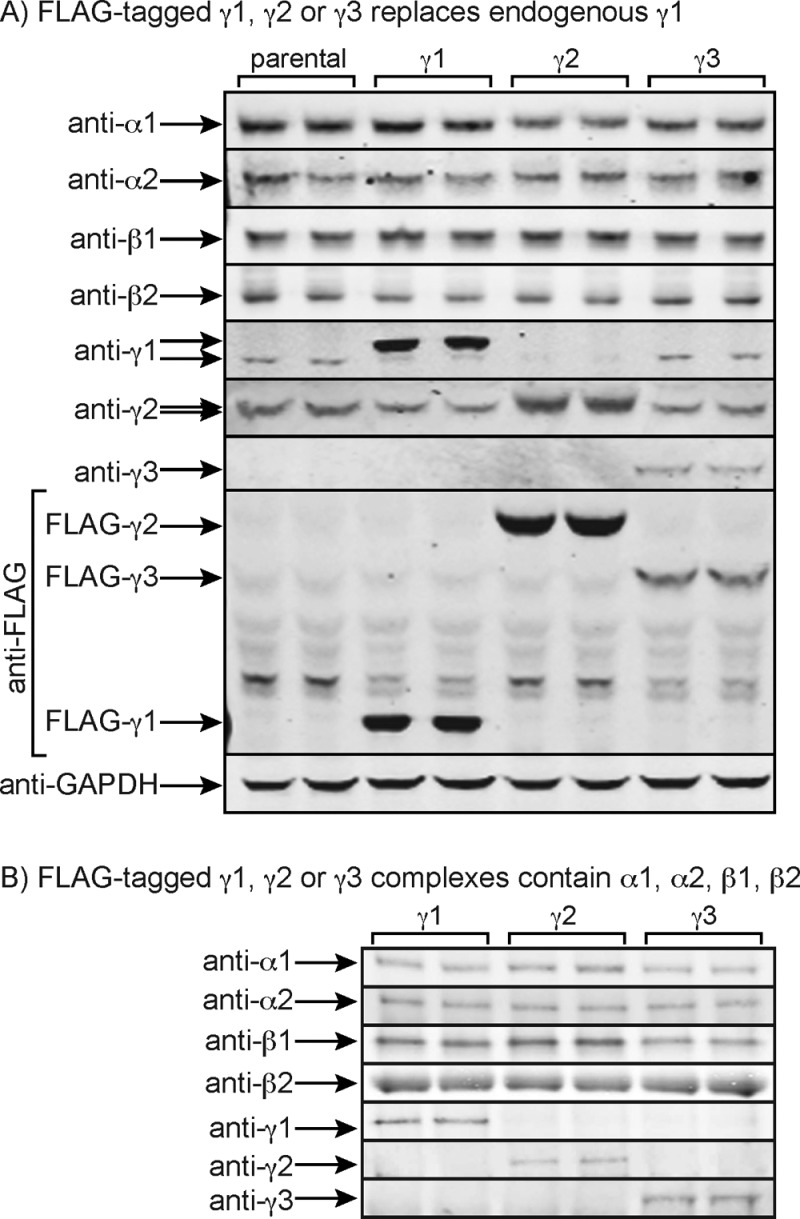
Characterization of HEK-293 cells stably expressing FLAG-tagged AMPK-γ1, -γ2 or -γ3 subunits and of immunoprecipitated complexes (**A**) Western blots of duplicate cultures of parental HEK-293 cells and cells expressing FLAG-tagged human AMPK-γ1, -γ2 or -γ3 subunits. Note that FLAG–γ1 has a slightly lower mobility on SDS/PAGE than the endogenous γ1; this effect is less obvious with endogenous γ2/FLAG–γ2 due to their higher molecular mass. The FLAG-tagged γ1, γ2 and γ3 subunits detected using the anti-FLAG antibody have markedly different mobilities due to their different molecular masses. (**B**) Western blots of duplicate IPs made using anti-FLAG antibodies from HEK-293 cells expressing FLAG-tagged human AMPK-γ1, -γ2 or -γ3 subunits. To correct for the different levels of expression of γ1, γ2 and γ3, 25, 15 and 50 μg respectively of lysate protein were loaded.

We first characterized the HEK-293 cells stably expressing FLAG-tagged human γ1, γ2 or γ3 by Western blotting. Figure 1(A) shows that, after tetracycline induction, the FLAG-tagged γ subunits partially, but not completely, replaced the endogenous γ1 subunit. The expression of endogenous γ1 (which migrates slightly faster on SDS/PAGE than FLAG–γ1) decreased in the cells expressing FLAG–γ1 and FLAG–γ2, although the effect was less obvious in the cells expressing FLAG–γ3, which was expressed at lower levels as judged by the anti-FLAG blots. The expression of endogenous γ2 was also slightly decreased in the cells expressing FLAG–γ1 and FLAG–γ3, although the expression of endogenous α1, α2, β1 or β2 did not change.

Coomassie Blue staining was not sufficiently sensitive to assess the composition of the γ1, γ2 or γ3 complexes that we immunoprecipitated using anti-FLAG antibodies, but we analysed them using Western blotting (Figure 1B). They each contained the expected γ subunit isoform, together with the same relative proportions of α1 and α2 and β1 and β2, suggesting that the different γ subunit isoforms did not show any selectivity in binding to α and β isoforms. The Western blotting data cannot be used to assess the absolute proportions of α1 compared with α2 or β1 compared with β2 in each complex. However, based on the AMPK activities of IPs made from the parental HEK-293 cells using anti-α1, -α2, -β1 and -β2 antibodies, we found that α1 contributed 95±0.2% and α2 contributed 5±0.2%, whereas β1 contributed 32±2% and β2 contributed 68±2%, of total kinase activity (means ± S.E.M., *n*=4). Thus, HEK-293 cells primarily express α1, whereas β1 contributes approximately one-third and β2 approximately two-thirds of total AMPK activity in these cells.

### AMPK-γ1, -γ2 and–γ3 complexes are all activated by Thr^172^ phosphorylation in intact cells

**Figure 2:**
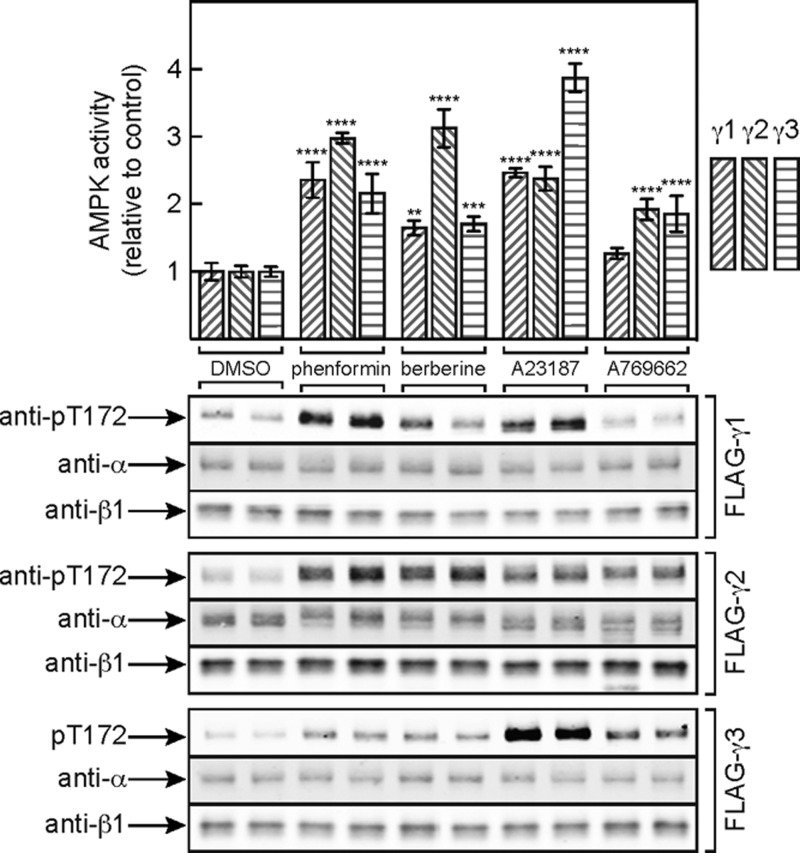
Activation of FLAG–γ1,–γ2 or–γ3 by various AMPK activators Cells expressing FLAG–γ1,–γ2 or–γ3 were incubated with DMSO (vehicle), phenformin (10 mM), berberine (300 μM), A23187 (10 μM) or A-769662 (300 μM) for 60 min, AMPK complexes were immunoprecipitated using anti-FLAG antibodies and were analysed by kinase assays (upper panel) and Western blotting (lower panel). In the upper panel, results are means ± S.D. (*n*=3); mean values significantly different from the DMSO control for each isoform (two-way ANOVA with Dunnett's multiple comparison test) are indicated: **P<0.01; ***P<0.001; ****P<0.0001. The absolute activities (nmol/min/mg of lysate protein) in the DMSO controls were: 0.16±0.01 (γ1), 0.24±0.01 (γ2) and 0.053±0.001 (γ3; means ± S.E.M., *n*=3).

Figure 2 shows that treatment with phenformin and another respiratory chain inhibitor, berberine, activated γ1-, γ2- and γ3-containing complexes and increased their phosphorylation on Thr^172^, with the largest effects being on the γ2 complex. The Ca^2+^ ionophore A23187, which activates the alternative upstream kinase CaMKKβ, also activated and caused Thr^172^ phosphorylation with all three complexes, although in this case the largest effects were on the γ3 complex (whose activation by A23187 was indeed mediated by CaMKKβ, because it was completely blocked by the CaMKK inhibitor STO-609 [50]; results not shown). By contrast, A-769662 caused only modest increases in AMPK activation and Thr^172^ phosphorylation with the γ2- and γ3-containing complexes and did not significantly activate γ1-containing complexes. The latter observation is similar to previous data obtained using rat hepatocytes (which express almost exclusively the γ1 isoform), where there was a marked phosphorylation of the downstream target acetyl-CoA carboxylase (ACC) despite the fact that any increases in Thr^172^ phosphorylation were negligible [51]. The lack of increased AMPK activation and Thr^172^ phosphorylation in response to A-769662, which binds at a different site from AMP [18,51], suggests that its major effect is on allosteric activation, with only a small secondary effect on Thr^172^ dephosphorylation.

### Allosteric activation of AMPK complexes containing γ1, γ2 or γ3

**Figure 3:**
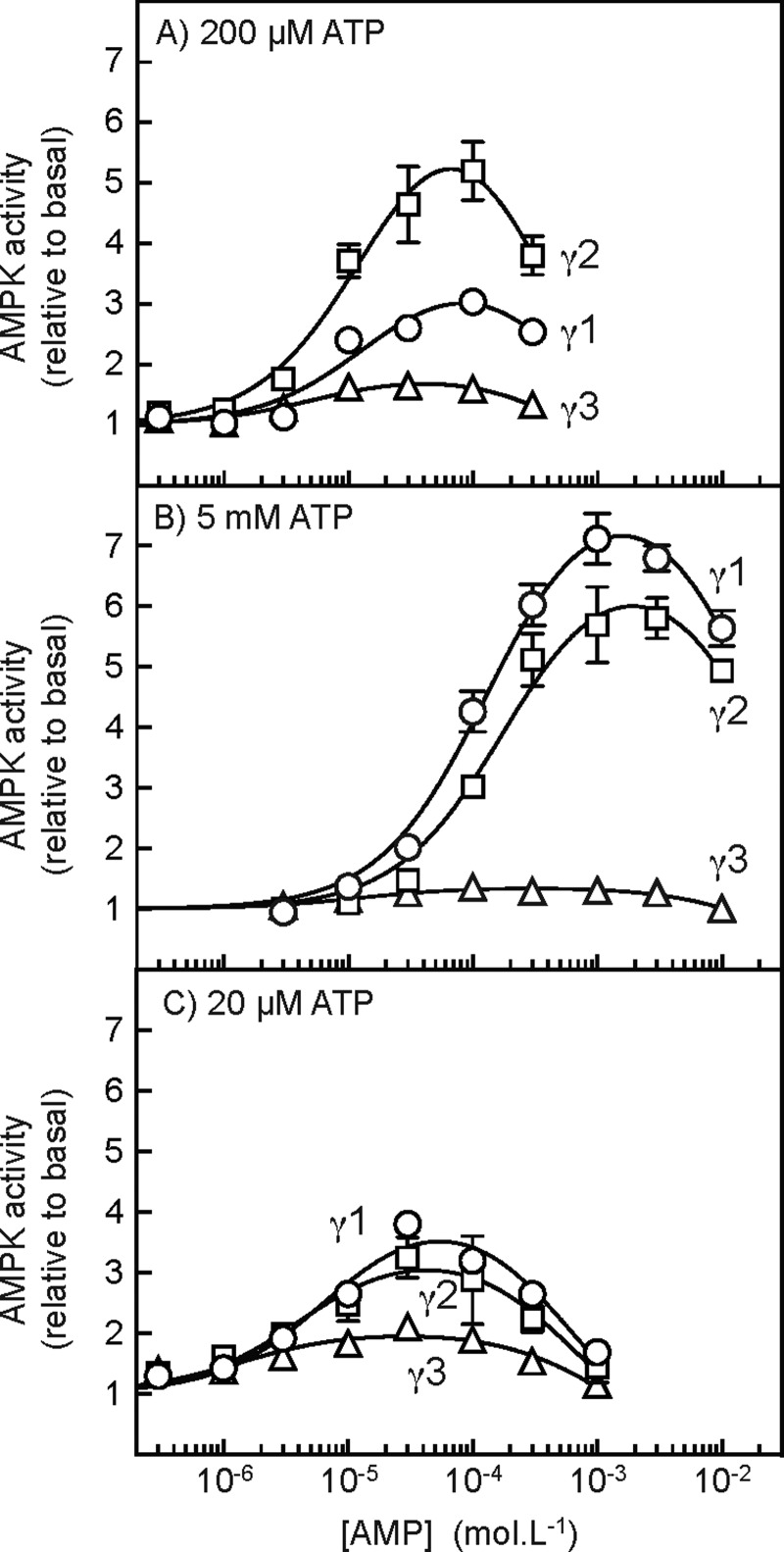
Allosteric activation of AMPK-γ isoforms at different concentrations of ATP Cells expressing FLAG–γ1,–γ2 or–γ3 were treated with 10 mM phenformin for 60 min to activate AMPK and the different complexes were immunoprecipitated using anti-FLAG antibody. Kinase assays were conducted at the indicated concentrations of AMP, but with ATP at 200 μM (**A**), 5 mM (**B**) or 20 μM (**C**). MgCl_2_ was also present at a concentration 4.8 mM higher than ATP; this protocol results in the proportions present as free ATP and as the MgATP^2−^ complex remaining approximately constant [55]. Results (means ± S.E.M., *n*=3) are expressed relative to the basal activity without AMP and were fitted to the equation: *Y*=1+{[(activation–1) × *X*]/(EC_50_+*X*)}–{[(activation) × *X*]/(IC_50_+*X*)}, where *Y* is the activity, *X* is the concentration of AMP, activation is the predicted maximal activation for the activating phase of the curve, EC_50_ is the concentration of AMP causing half-maximal activation, and IC_50_ is the concentration of AMP causing half-maximal inhibition. The continuous lines are the curves generated by this equation using the values for activation, EC_50_ and IC_50_ quoted in the text.

We next addressed the allosteric activation of complexes containing the different γ isoforms, using AMPK immunoprecipitated from HEK-293 cell stably expressing FLAG-tagged γ1, γ2 or γ3. These assays were initially performed both at the standard (but sub-physiological) ATP concentration of 200 μM (Figure 3A) and at the more physiological ATP concentration of 5 mM (Figure 3B). In all cases, the AMPK complexes were activated at low concentrations of AMP due to binding at the γ subunit and then inhibited at higher concentrations due to competition with ATP at the catalytic site on the α subunit. However, when the assays were performed at 5 mM rather than 200 μM ATP both the activating and the inhibiting phases of the curves were shifted to the right by more than one order of magnitude (compare Figures 3A and 3B). These findings mirror our previous findings using rat liver AMPK (a mixture of the α1β1γ1 and α2β1γ1 isoforms) and are consistent with the idea that ATP antagonizes the effect of AMP not only at the site(s) on the γ subunit where it causes allosteric activation, but also at the catalytic site where (at higher concentrations) AMP causes inhibition [28]. Fitting the data to an equation assuming a single activating site and a single inhibitory site yielded the following parameters (means ± S.E.M.) with assays performed at 200 μM ATP: γ1 complex, activation, 3.9±0.5-fold; concentration of AMP giving half-maximal activation (EC_50_) 15±5 μM; concentration of AMP giving half-maximal inhibition (IC_50_) 0.66±0.28 mM; γ2 complex, activation, 7.8±1.0-fold; EC_50_, 16±4 μM; IC_50_, 0.34±0.09 mM; γ3 complex, activation, 1.9±0.1-fold; EC_50_, 5.5±2.1 μM; IC_50_, 0.73±0.22 mM. Thus, at 200 μM ATP, activation was greatest with the γ2 complex (8-fold), was less with the γ1 complex (4-fold) and was smallest (1.9-fold) with the γ3 complex. When the assays were conducted with 5 mM ATP the following parameters were obtained: γ1 complex, activation, 10±2-fold; EC_50_, 120±20 μM; IC_50_, 22±5 mM; γ2 complex, activation, 9±1-fold; EC_50_, 160±30 μM; IC_50_, 29±6 mM; γ3 complex, activation, 1.4±0.03-fold; EC_50_, 12±5 μM; IC_50_, 29±6 mM. Thus, when measured at this more physiological concentration of ATP, allosteric activation by AMP of both the γ1 and the γ2 complexes increased to ∼10-fold, whereas activation of the γ3 complex became almost negligible (1.4-fold). In addition, the EC_50_ values for allosteric activation by AMP of the γ1 and γ2 complexes were ∼10-fold higher at the higher ATP concentration (120 and 160 μM at 5 mM ATP, compared with 15 and 16 μM at 200 μM ATP), whereas the IC_50_ values for inhibition were 30–100-fold higher (22 and 29 mM at 5 mM ATP, compared with 0.66 and 0.34 mM at 200 μM ATP). This reflects the fact, as shown previously with rat liver AMPK [28], that AMP and ATP compete for binding not only at the binding site(s) on the γ subunits that cause allosteric activation, but also at the catalytic site where AMP binding causes inhibition.

Since there was more activation of the γ3 complex at the lower ATP concentration (200 μM), we repeated the assays in the presence of ATP concentrations 10-fold lower again (20 μM). Under these conditions (Figure 3C), maximal activation of the γ1 and γ2 complexes was lower than it was at higher concentrations of ATP, but maximal activation of the γ3 complex increased, although it was still significantly less than that of the other two. Fitting of the data yielded the following parameters: γ1, activation, 4.2±1.3-fold; EC_50_, 7.0±1.3 μM; IC_50_, 0.58±0.09 mM; γ2, activation, 3.5±0.2-fold; EC_50_, 4.2±1.2 μM; IC_50_, 0.65±0.15 mM; γ3, activation, 2.0±0.1-fold; EC_50_, 1.4±0.5 μM; IC_50_, 1.2±0.3 mM.

### Allosteric activation of bacterially expressed AMPK complexes

**Figure 4:**
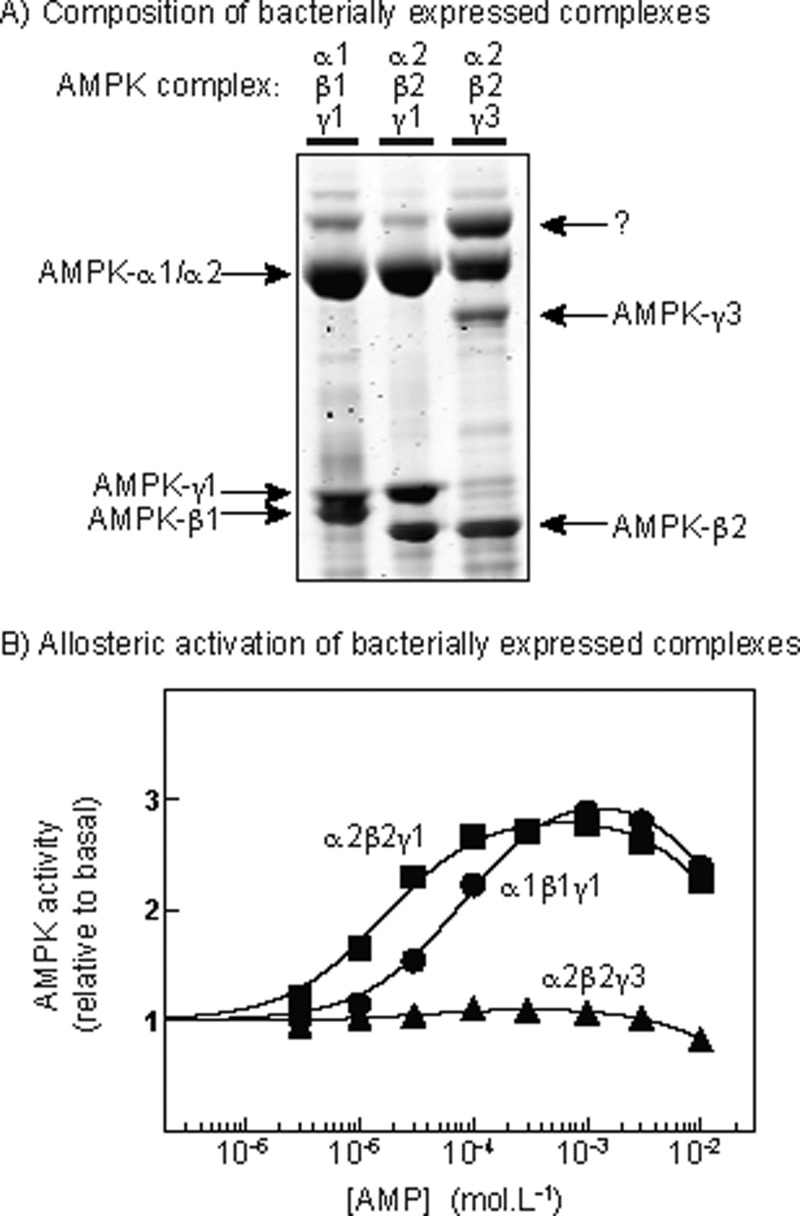
Composition and allosteric activation of bacterially expressed AMPK complexes (**A**) Analysis of α1β1γ1, α2β2γ1 and α2β2γ3 complexes by SDS/PAGE and Coomassie Blue staining; (**B**) allosteric activation of α1β1γ1, α2β2γ1 and α2β2γ3 complexes by AMP. Assays in (**B**) were performed in the presence of 5 mM ATP and 9.8 mM MgCl_2_ and the data fitted to the same equation as in Figure 3. *Note added in proof*: The band marked “?” in (**A**) has been identified by mass fingerprinting to be the bacterial protein arnA (UNIPROT P77398).

In order to confirm our results using complexes with a more well-defined isoform composition, we expressed the human α1β1γ1, α2β2γ1 and α2β2γ3 complexes in bacteria, purified them on Ni^2+^–Sepharose and phosphorylated Thr^172^ using CaMKKβ. Figure 4(A) shows Coomassie Blue-stained gels of these preparations, whereas Figure 4(B) shows that, when assayed at 5 mM ATP, the α1β1γ1 and α2β2γ1 complexes were allosterically activated to similar extents by AMP, although activation was more potent for the α2β2γ1 complex (α2β2γ1: activation, 2.9±0.1-fold; EC_50_, 16±1 μM; α1β1γ1: activation, 3.2±0.1-fold; EC_50_, 83±7 μM). However, the α2β2γ3 complex was barely activated at all (<15%) by AMP; this is similar to the results we obtained with γ1 and γ3 complexes expressed in HEK-293 cells (Figure 3), except that maximal activation by AMP was much smaller with the bacterially expressed γ1 complexes (∼3-fold) compared with those expressed in mammalian cells (9–10-fold). We have observed this difference consistently, which it is why we used the mammalian expression system for the remaining studies described in the present paper.

### Promotion of LKB1-dependent activation and Thr^172^ phosphorylation by AMP

**Figure 5:**
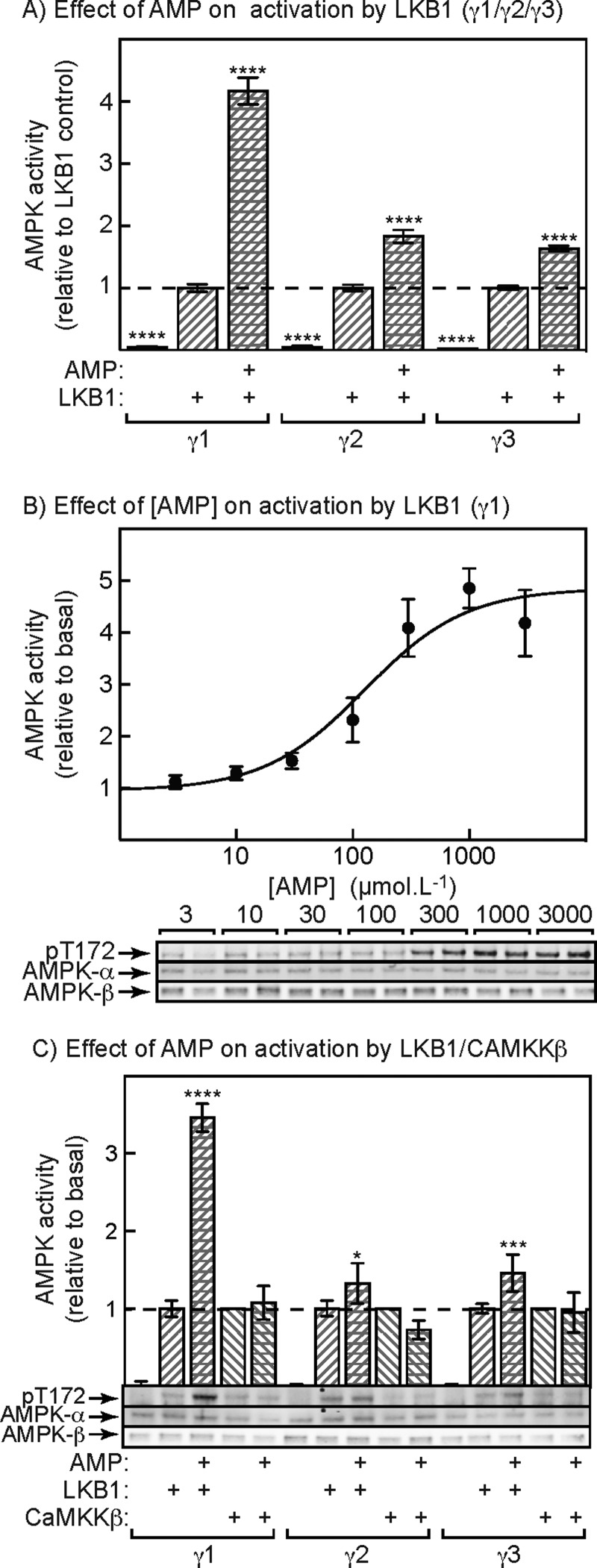
Effect of AMP on activation and Thr^172^ phosphorylation of AMPK-γ isoforms induced by upstream kinases (**A**) Effect of a limiting quantity of LKB1, with or without AMP (1 mM), on AMPK activity. Anti-FLAG IPs from AMPK-γ1-, -γ2- or -γ3-expressing cells were treated with PP1γ to bring about complete dephosphorylation of Thr^172^. PP1γ was removed by washing and okadaic acid was added to ensure complete inhibition of any residual phosphatase. The IPs were then incubated for 10 min with 5 mM ATP with or without LKB1 (1 μg) and with or without 1 mM AMP. Results are means ± S.E.M. (*n*=6) and mean values significantly different from controls with LKB1 but without AMP (two-way ANOVA with Dunnett's multiple comparison test) are indicated (****P<0.0001). (**B**) Effect of different concentrations of AMP on activation and Thr^172^ phosphorylation of the AMPK-γ1 complex. Experiment as in (**A**) except using various concentrations of AMP in the presence of 5 mM ATP. Results in the upper panel (means ± S.E.M., *n*=6) are expressed relative to the control without AMP. Results were fitted to the equation: *Y*=1+[(activation–1) × *X*]/(EC_50_+*X*), where *Y* is the relative activity, *X* is the concentration of AMP, activation is the predicted maximal activation, and EC_50_ is the concentration of AMP causing half-maximal activation. The continuous line is the curve generated using this equation with the parameters quoted in the text. The lower panel shows duplicate Western blots from the same experiment. (**C**) As (**A**), except that we also tested the effects of 1 mM AMP on activation by CaMKKβ as well as LKB1. The amount of CaMKKβ added (0.34 μg) yielded a similar limited degree of activation to LKB1. Results significantly different from the controls without AMP, by two-way ANOVA with Tukey's multiple comparison test, are indicated: *P<0.05, ***P<0.001, ****P<0.0001. The lower panel shows Western blots of single samples from the same experiment.

We next tested whether AMP promoted Thr^172^ phosphorylation by LKB1, using complexes containing γ1, γ2 or γ3 expressed in HEK-293 cells and isolated using anti-FLAG antibody. The IPs were treated with the protein phosphatase-1 catalytic subunit (PP1γ) to remove phosphate from Thr^172^, were washed to remove PP1γ, and okadaic acid was added to block the activity of any residual phosphatase. They were then treated with limiting quantities of LKB1 in the presence of 5 mM ATP (to mimic physiological conditions), with or without 1 mM AMP. This revealed (Figure 5A) that AMP caused a 4-fold stimulation of activation of the γ1 complex, whereas the effects of AMP on the γ2 and γ3 complexes were much smaller (<2-fold), although still significant. Figure 5(B) shows the promotion of activation and Thr^172^ phosphorylation of the γ1 complex by LKB1 at various concentrations of AMP, once again with 5 mM ATP in the assays. Maximal activation was estimated to be 5.1±1.3-fold and the half-maximal effect of AMP was at 130±50 μM, very similar to the EC_50_ obtained for allosteric activation by AMP under the same conditions (120±20 μM).

We also tested whether AMP promoted AMPK activation and Thr^172^ phosphorylation by CaMMKβ as well as LKB1, as proposed previously [29]. The experiment was similar to that shown in Figure 5(A) except that we also used a limiting amount of CaMKKβ that gave a similar degree of Thr^172^ phosphorylation and activation to that of LKB1. Figure 5(C) shows that, using LKB1 as the upstream kinase, AMP caused a substantial promotion of AMPK activation as well as Thr^172^ phosphorylation, with smaller effects on the γ2 and γ3 complexes, as in Figure 5(A). However, AMP did not promote AMPK activation or Thr^172^ phosphorylation by CaMKKβ using any of the γ subunit isoforms.

### Promotion of LKB1-dependent activation and Thr^172^ phosphorylation by ADP

**Figure 6:**
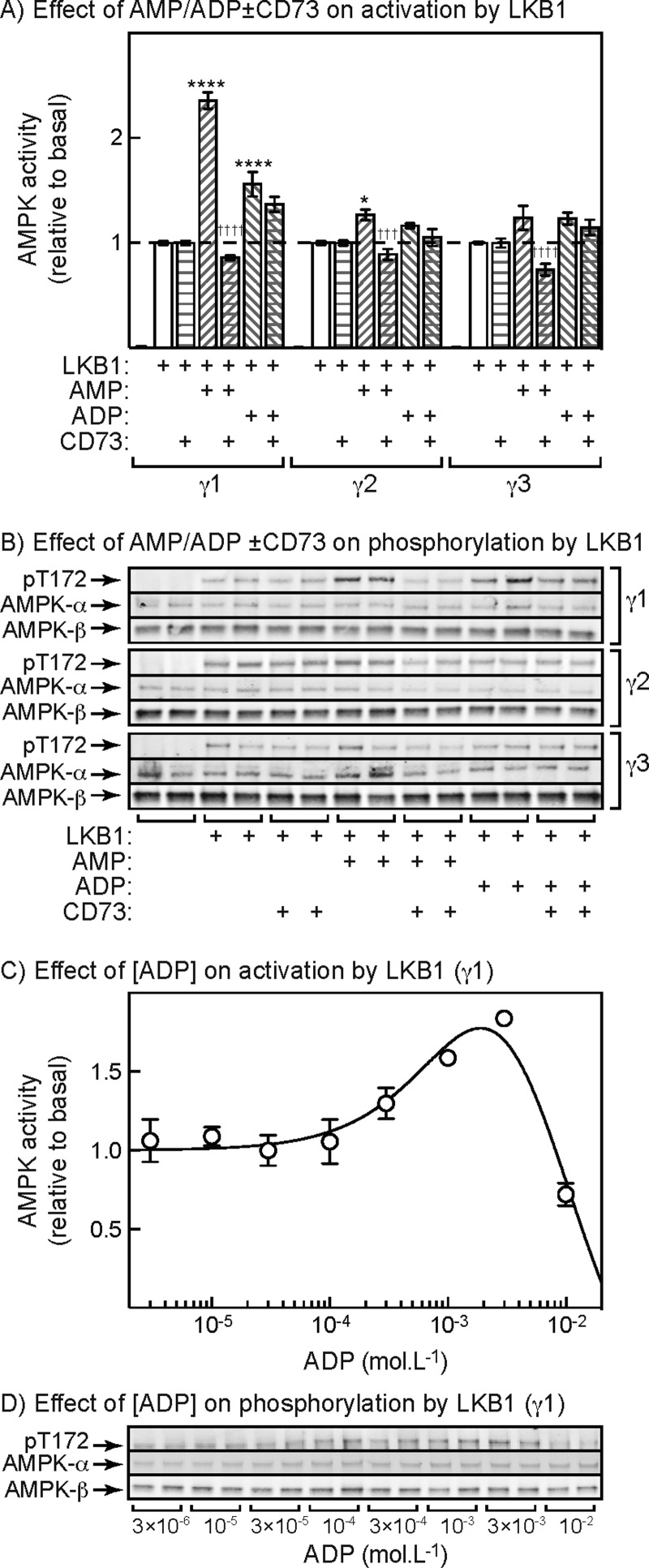
Effect of ADP on activation and Thr^172^ phosphorylation of AMPK-γ isoforms induced by upstream kinases (**A**) Effect of AMP or ADP on activation of γ1-, γ2- or γ3-containing AMPK complexes induced by LKB1 in the presence of 5 mM ATP. Dephosphorylated AMPK complexes were generated as in Figure 3(A) and were incubated for 10 min with LKB1 with or without AMP (300 μM), ADP (1 mM) or the 5′-nucleotidase CD73 (0.86 μg). Results are means ± S.E.M. (*n*=4) and results significantly different with/without AMP or ADP (*P<0.05; ****P<0.0001) and with/without CD73 (^†††^*P*<0.001, ^††††^*P*<0.0001), determined by two-way ANOVA with Tukey's multiple comparison test, are indicated. (**B**) Duplicate blots from the experiment shown in (**A**). (**C**) Effect of ADP on activation of AMPK-γ1 complexes by LKB1 in the presence of 5 mM ATP. The experiment was performed as in (**A**) except with various concentrations of ADP. Results (means ± S.E.M., *n*=3) are expressed relative to the basal activity in the absence of ADP and were fitted to the equation used for Figure 3(B). The continuous line is the curve generated using the best-fit parameters (activation=5-fold, EC_50_=1.8±1.1 mM; IC_50_=6.8±3.1 mM; activation was constrained to be 5-fold or less). However, the confidence limits for these parameters are extremely wide because the activating and inhibitory phases of the curve are so close together. (**D**) Duplicate blots obtained from the experiment shown in (**C**).

Using native AMPK purified from rat liver (a mixture of the α1β1γ1 and α2β1γ1 complexes), we previously reported small effects of ADP on activation and Thr^172^ phosphorylation by LKB1 [28]. However, we suspected at that time that this was due to breakdown of ADP to AMP during the assay, because the effect of ADP appeared to be abolished by addition of the 5′-nucleotidase, CD73, which degrades AMP but not ADP. We addressed this question again using our immunoprecipitated human γ1, γ2 and γ3 complexes (Figure 6). As before, AMP gave a large stimulation of activation and Thr^172^ phosphorylation with the γ1 complex and smaller effects with the γ2 and γ3 complex and all of these effects were abolished by the inclusion of CD73 in the assays as expected. ADP also appeared to cause a small stimulatory effect with the γ1 complex, although any effects on the γ2 and γ3 complexes were not significant. Unexpectedly, however, CD73 did not completely abolish the small stimulatory effect of ADP on the γ1 complex, suggesting that the effect was at least partly caused by binding of ADP, rather than by breakdown of ADP to AMP during the assay. The Western blots in Figure 6(B) confirm that the stimulatory effect of AMP on Thr^172^ phosphorylation induced by LKB1 was completely abolished by addition of CD73, whereas the stimulatory effect of ADP was only partly abolished. Any effects of ADP on Thr^172^ phosphorylation by LKB1 of the γ2 and γ3 complexes were not detectable, although there were small effects of AMP that were abolished by CD73.

Figure 6(C) shows the concentration dependence for the effect of ADP on activation by LKB1 using the γ1 complex, whereas Figure 6(D) shows Thr^172^ phosphorylation assessed by Western blotting in the same experiment. Concentrations of ADP from 0.1 to 1 mM caused a small stimulatory effect, but above 1 mM ADP caused a marked inhibition. It was not possible to obtain precise estimates of the EC_50_ for the activating effect or the IC_50_ for the inhibitory effect, because the ranges of concentrations over which they occurred were so similar. However, the EC_50_ for the activating effect of ADP appeared to be at least 1 mM, which is an order of magnitude higher than the EC_50_ for the effect of AMP (130 μM).

### Protection against dephosphorylation of Thr^172^ by AMP and ADP

**Figure 7:**
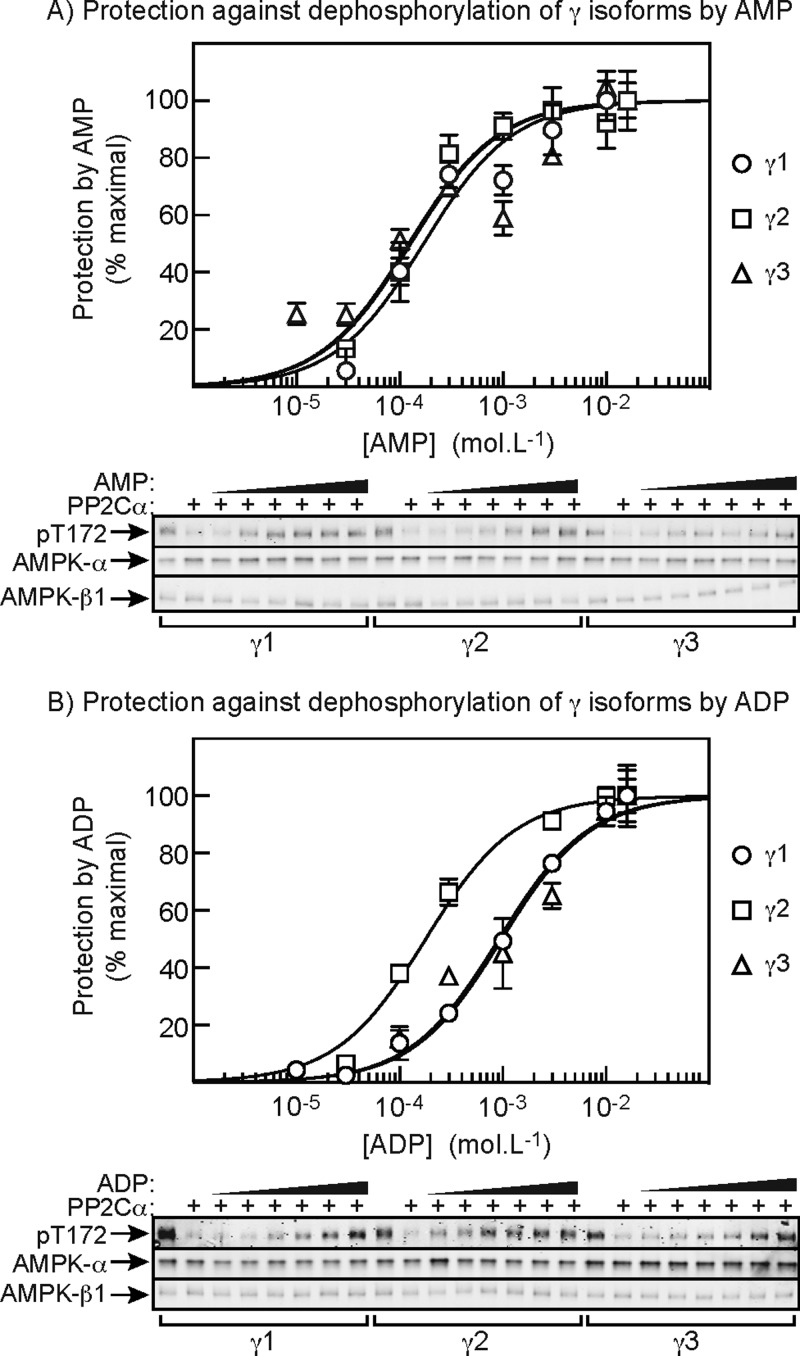
Effect of AMP and ADP on protection against Thr^172^ dephosphorylation, measured at 5 mM ATP (**A**) AMP-γ1, -γ2 or -γ3 complexes were immunoprecipitated from cells treated with phenformin to cause maximal Thr^172^ phosphorylation. The IPs were then incubated for 10 min with the protein phosphatase PP2Cα in the presence of 5 mM ATP and various concentrations of AMP. Inactivation was assessed by kinase assays (top panel) and Thr^172^ dephosphorylation by Western blot (bottom panels). Results (means ± S.E.M., *n*=2) in the top panel are shown as percentage protection, which is the inactivation compared with control (without AMP), expressed as a percentage of the inactivation compared with control at the highest concentration of AMP used. Results were fitted to the equation *Y*=100 × *X*/(EC_50_+*X*) where *Y* is the percentage protection, *X* is the concentration of AMP, and EC_50_ is the concentration of AMP causing half-maximal protection. The continuous lines are the curves generated using this equation using the best-fit parameters quoted in the text. The lower panel shows single Western blot blots for each condition, with the increasing concentrations of AMP being 30 μM, 100 μM, 300 μM, 1 mM, 3 mM and 10 mM. (**B**) As (**A**), but varying ADP in place of AMP.

Finally, we addressed the effects of AMP and ADP on the dephosphorylation of γ1, γ2 and γ3 complexes by the protein phosphatase PP2Cα. Cells expressing γ1, γ2 or γ3 complexes were treated with phenformin to promote Thr^172^ phosphorylation, the complexes were then isolated by immunoprecipitation and equal amounts (based on anti-FLAG blots) were treated with a limiting quantity of PP2Cα in the presence of increasing concentrations of AMP or ADP. As before, in order to simulate physiological conditions, these assays were performed in the presence of 5 mM ATP. The upper panel of Figure 7(A) shows that inactivation of all three complexes was protected in a very similar manner by AMP, with the half-maximal effects occurring at 170±30 (γ1), 130±20 (γ2) and 120±30 μM (γ3). The similar effects of AMP on all three complexes were confirmed by Western blot analysis using anti-pThr^172^ antibodies (lower panel). The effects of ADP are shown in Figure 7(B); similar to results previously obtained with rat liver AMPK [28] (which contains exclusively the γ1 isoform), with the γ1 and γ3 complexes we found that the curves were shifted rightward, compared with those for AMP, by almost one order of magnitude. This indicates that ADP has a less potent protective effect than AMP, which was reflected in the IC_50_ values: 170±30 μM (AMP) and 910±70 (ADP) for γ1 and 120±30 μM (AMP) and 980±150 (ADP) for γ3. However, a striking difference with the γ2 complex was that ADP was almost equally potent in protecting against inactivation and Thr^172^ dephosphorylation as AMP. In this case, the IC_50_ values were 130±20 μM (AMP) and 180±20 (ADP). The upper panel of Figure 7(B) shows that the curve for protection of γ2 complexes by ADP was clearly different from those for the γ1 and γ3 complexes and this difference can also be discerned in the anti-pThr^172^ blots (lower panel).

## DISCUSSION

Our results clearly demonstrate that human AMPK complexes containing the γ1, γ2 or γ3 isoforms, when expressed in human cells, differ in several respects in their responses to adenine nucleotide. These differences can be summarized as follows:

(i) Complexes containing γ1 and γ2 were allosterically activated by AMP up to 10-fold, with half-maximal effects (measured in the presence of 5 mM ATP) at 120 (γ1) and 160 μM (γ2), which are 30–40-fold lower than the concentration of ATP utilized in the assay (5 mM). By contrast, γ3 complexes were only significantly allosterically activated by AMP (2-fold) at very low ATP concentrations (20 μM) that are unlikely to be physiologically relevant.
(ii) Activation of γ1 complexes caused by Thr^172^ phosphorylation was stimulated >4-fold by AMP when LKB1, but not CaMKKβ, was the upstream kinase. Much smaller (<2-fold), although still significant, effects of AMP were observed using γ2 and γ3 complexes. The half-maximal effect of AMP on activation of γ1 complexes by LKB1, measured at 5 mM ATP (130 μM), was very similar to the half-maximal effect for allosteric activation (120 μM).
(iii) Activation of γ1 complexes caused by Thr^172^ phosphorylation by LKB1 was also stimulated ∼2-fold by ADP, although we did not observe this effect with γ2 or γ3 complexes. Despite our earlier findings with native AMPK from rat liver [28], this effect of ADP could not be completely explained by its breakdown in the assay to AMP, because it was only partially abolished by addition of the 5′-nucleotidase CD73. However, the effect did require ADP concentrations an order of magnitude higher than AMP and at concentrations only slightly higher again, ADP markedly inhibited activation.
(iv) Inactivation of all three γ complexes by Thr^172^ dephosphorylation, catalysed by PP2Cα, was reduced by AMP binding. The concentrations of AMP causing half-maximal effects, measured at 5 mM ATP, were similar for all three complexes (120–170 μM) and were similar to the concentrations causing half-maximal allosteric activation with γ1 and γ2 complexes and half-maximal activation of phosphorylation/activation by LKB1 using γ1 complexes. All of these effects were measured in the presence of a physiologically relevant concentration of ATP (5 mM).
(v) Inactivation of all three γ complexes by Thr^172^ dephosphorylation catalysed by PP2Cα was also protected by binding of ADP. Interestingly, however, the concentrations causing half-maximal effects, measured at 5 mM ATP, were similar for AMP and ADP using γ2 complexes (130 and 180 μM), but those for ADP were almost an order of magnitude higher than those for AMP using γ1 and γ3 complexes. Thus AMP is in general a more potent activator of AMPK than ADP, with the interesting exception of the γ2 complex.

The AMPK activity in HEK-293 cells is largely accounted for by the α1, β1, β2 and γ1 isoforms [49], although in the cells we studied the expressed γ1, γ2 and, to a lesser extent, γ3 subunits replaced the endogenous γ1 (Figure 1A). Complexes containing endogenous γ1 would not have been present in our IPs because they would not be FLAG-tagged. However, we chose not to study effects on any downstream targets such as ACC1 in the intact cells, because such results might have been compromised by the presence in the cells of complexes containing endogenous γ1. The complexes isolated from HEK-293 cells would have predominantly been the α1β2γ1, α1β2γ2 or α1β2γ3 complexes, although the anti-FLAG IPs also contained some α2 and β1 and there may have been minor contributions from those isoforms. It was not feasible to confirm the purity of the complexes in the IPs by Coomassie Blue staining due to its lack of sensitivity, so we cannot exclude the possibility that other proteins associating with the AMPK heterotrimer might have influenced the results. However, the results obtained with the bacterially expressed α1β1γ1, α2β2γ1 and α2β2γ3 complexes in Figure 4(B) suggest that it is indeed the identity of the γ subunit isoform that determines the allosteric response to AMP, in that the α1β1γ1 and α2β2γ1 complexes were clearly activated by AMP when the assays were performed in the presence of 5 mM ATP, whereas activation of the α2β2γ3 complex was minimal. The α2β2γ3 complex is the only complex found to be activated by contraction in skeletal muscle, although since activation was measured after immunoprecipitation [8], it must have been due to increased Thr^172^ phosphorylation rather than any allosteric effect. It is indeed notable that the complex that is activated by muscle contraction is not subject to allosteric activation.

It remains unclear as to why the allosteric activation by AMP of γ1 and γ2 complexes is much greater for those expressed in mammalian cells rather than in bacteria, although we suspect that it may be due to some covalent modification (other than Thr^172^ phosphorylation) that occurs in mammalian cells but not in bacteria. Since the regulation of the bacterially expressed complexes is in that sense abnormal, we chose to study the other regulatory mechanisms only with the complexes expressed in human cells.

In 2011, it was reported that ADP was almost as effective as AMP in promoting Thr^172^ phosphorylation on AMPK, using CaMKKβ as the upstream kinase [29]. Using native AMPK purified from rat liver (a mixture of the α1β1γ1 and α2β1γ1 complexes) we reported that ADP did stimulate activation and Thr^172^ phosphorylation by LKB1, but not by CaMKKβ. However, we suspected that this effect of ADP was caused by its breakdown in the assay to AMP, because it appeared to be abolished by the addition of CD73, a 5′-nucleotidase that hydrolyses AMP to adenosine, but has no effect on ADP. In the present study, we observed small effects of activation and Thr^172^ phosphorylation of γ1 complexes by ADP and these effects were reduced but not abolished by CD73. The CD73 was clearly functional in the assay because it did abolish the effects of AMP (Figures 6A and 6B). The effects of ADP could not therefore be entirely explained by breakdown of ADP to AMP in the assay. Despite this, we suspect that the stimulatory effect of ADP on activation of γ1 complexes might not have much physiological relevance. The effect is only observed with γ1 complexes, is small (<2-fold) and requires high concentrations of ADP (>1 mM). Moreover, at concentrations of ADP only slightly higher again (10 mM), ADP caused a marked inhibition to well below the basal activity (Figure 6C and 6D). At this concentration, ADP is higher than ATP in the assay and it may simply inhibit the upstream kinase LKB1 by competing with ATP at the catalytic site. Thus, there is only a narrow window of concentrations where ADP might have a stimulatory effect on Thr^172^ phosphorylation by LKB1.

What is the reason for the discrepancy between our results, in which AMP binding did not promote activation and Thr^172^ phosphorylation of γ1 complexes by CaMKKβ and those of others who reported that AMP does promote activation of an α1β1γ1 complex (expressed in COS7 cells) by CaMKKβ [29]? One possible explanation is that in the latter study the α1β1γ1 complex had been expressed with a GST-tag on the α1 subunit. The complex was dephosphorylated on glutathione–Sepharose beads using recombinant PP2Cα and the “beads were washed extensively before elution” prior to performing the phosphorylation experiment. However, the PP2Cα used, which was expressed in Sf9 insect cells, had also been GST-tagged and was purified on glutathione–Sepharose [52]. Although the GST tag was “completely removed from PP2C by tobacco etch virus protease treatment” if there was any residual uncleaved GST-tag or the washing did not remove all of the phosphatase, then their final preparation of dephosphorylated AMPK might have been contaminated with PP2Cα. The apparent effects of AMP to promote Thr^172^ phosphorylation might then have been caused by the ability of AMP to inhibit dephosphorylation. We avoided this potential problem by dephosphorylating AMPK in the IP using PP1γ rather than PP2Cα, by washing the precipitates extensively and by adding the PPP family phosphatase inhibitor, okadaic acid [53], to the phosphorylation reaction to inhibit any traces of residual PP1γ.

The molecular mechanisms for the differential effects of AMP, ADP and ATP on AMPK complexes containing the γ1, γ2 and γ3 isoforms remain unclear at present. All existing crystal structures for core [24,26] or full-length [18,20,27,54] heterotrimers with bound adenine nucleotides contain only the γ1 isoform and the exact roles of the three nucleotide-binding sites in the three mechanisms of regulation remain poorly understood. The sequences of the CBS repeats in the γ1, γ2 and γ3 isoforms are highly conserved, but it seems possible that subtle differences in the affinities of sites 1, 3 and 4 for AMP, ADP and ATP could explain the differences in regulation reported in the present study. The major differences in the sequences of the γ subunit isoforms lie in their N-terminal regions, with γ2 and γ3 having N-terminal extensions of approximately 250 and 180 residues respectively, which are unrelated to each other and are not present in γ1; both γ2 and γ3 also occur as shorter variants [35,36]. Although the functions of these N-terminal extensions remain uncertain, there is evidence that different isoform combinations are found at different subcellular locations [30,35,37] and it seems possible that the N-terminal extensions are involved in targeting of AMPK to specific subcellular locations. The only cell type that clearly expresses all three isoforms of the γ subunit is skeletal muscle, where compartmentation may be a particular issue due to the abundance of the contractile machinery. It is tempting to propose that the differential responses to AMP, ADP and ATP of AMPK complexes containing distinct γ subunit isoforms may allow the fine-tuning of responses to energy stress in specific subcellular locations, especially in muscle. However, any detailed discussion of the potential physiological role of these findings would only be speculative at present.

## Abbreviations

ACC: acetyl-CoA carboxylase (ACC)
AICAR: 5-aminoimidazole-4-carboxamide riboside
AMPK: AMP-activated protein kinase
CaMKK: calmodulin-dependent kinase kinase
CBM: carbohydrate-binding module
CBS: cystathionine-β-synthase
IP: immunoprecipitate
LKB1: liver kinase B1
PP1γ: protein phosphatase-1 catalytic subunit
